# Variety of genetic defects in GnRH and hypothalamic–pituitary signaling and development in normosmic patients with IHH

**DOI:** 10.3389/fendo.2024.1396805

**Published:** 2024-07-01

**Authors:** Małgorzata Kałużna, Bartłomiej Budny, Michał Rabijewski, Agnieszka Dubiel, Małgorzata Trofimiuk-Müldner, Kosma Szutkowski, Adam Piotrowski, Elżbieta Wrotkowska, Alicja Hubalewska-Dydejczyk, Marek Ruchała, Katarzyna Ziemnicka

**Affiliations:** ^1^ Department of Endocrinology, Metabolism and Internal Diseases, Poznan University of Medical Sciences, Poznan, Poland; ^2^ Department of Reproductive Health, Centre for Postgraduate Medical Education, Warsaw, Poland; ^3^ Chair and Department of Endocrinology, Jagiellonian University Medical College, Kraków, Poland; ^4^ NanoBioMedical Centre at Adam Mickiewicz University in Poznan, Poznan, Poland; ^5^ Department of Biomedical Physics at Adam Mickiewicz University in Poznan, Poznan, Poland

**Keywords:** isolated hypogonadotropic hypogonadism, oligogenicity, next-generation sequencing (NGS), pathogenic/likely pathogenic (P/LP), normosmic

## Abstract

**Introduction:**

Normosmic isolated hypogonadotropic hypogonadism (nIHH) is a clinically and genetically heterogeneous disorder. Deleterious variants in over 50 genes have been implicated in the etiology of IHH, which also indicates a possible role of digenicity and oligogenicity. Both classes of genes controlling GnRH neuron migration/development and hypothalamic/pituitary signaling and development are strongly implicated in nIHH pathogenesis. The study aimed to investigate the genetic background of nIHH and further expand the genotype–phenotype correlation.

**Methods:**

A total of 67 patients with nIHH were enrolled in the study. NGS technology and a 38-gene panel were applied.

**Results:**

Causative defects regarded as at least one pathogenic/likely pathogenic (P/LP) variant were found in 23 patients (34%). For another 30 individuals, variants of unknown significance (VUS) or benign (B) were evidenced (45%). The most frequently mutated genes presenting P/LP alterations were *GNRHR* (*n* = 5)*, TACR3* (*n* = 3), and *CHD7, FGFR1, NSMF, BMP4*, and *NROB1* (*n* = 2 each). Monogenic variants with solid clinical significance (P/LP) were observed in 15% of subjects, whereas oligogenic defects were detected in 19% of patients. Regarding recurrence, 17 novel pathogenic variants affecting 10 genes were identified for 17 patients. The most recurrent pathogenic change was *GNRHR*:p.Arg139His, detected in four unrelated subjects. Another interesting observation is that P/LP defects were found more often in genes related to hypothalamic–pituitary pathways than those related to GnRH.

**Conclusions:**

The growing importance of the neuroendocrine pathway and related genes is drawing increasing attention to nIHH. However, the underestimated potential of VUS variants in IHH etiology, particularly those presenting recurrence, should be further elucidated.

## 1 Introduction

Isolated hypogonadotropic hypogonadism (IHH) is a rare genetic disorder comprising gonadoliberin (GnRH) deficiency with anosmia or hyposmia [Kallmann syndrome (KS)] and normal olfaction known as normosmic IHH (nIHH). The prevalence of IHH is approximately 1–10 in 100,000 live births, with approximately 40% of cases being normosmic (1). Both KS and nIHH are clinically and genetically heterogeneous. The diagnosis of IHH is challenging since patients with nIHH occasionally present a wide variety of nonreproductive defects, such as midline facial defects, renal anomalies, or limb malformations (2–4). Deficiency of GnRH secretion or action results in the impairment of pubertal development. The clinical spectrum of male nIHH includes absent or incomplete puberty, micropenis, cryptorchidism, impaired libido and potency, and infertility. Primary or secondary amenorrhea, partial breast development and infertility may occur in the course of female nIHH (2–4). Neonatal presentation of nIHH with a lack of mini-puberty and abnormal or underdeveloped external genitalia could be overlooked (5). Diagnostic difficulties in the population classified as nIHH may also be caused by nongenetic conditions that may affect GnRH secretion (6–9). The genetic background overlap seen for KS, nIHH, combined pituitary hormone deficiency (CPHD), and hypothalamic amenorrhea hinders routine genetic diagnostics for hypogonadism (10–13). The dysfunction of crucial components of the GnRH pathway underlies the pathogenesis of IHH (14, 15). Deleterious variants in over 50 genes contributing to neurodevelopmental or neuroendocrine activities have been implicated in the etiology of IHH (13, 15, 16). An abundance of genetic factors can impact heritability, leading to autosomal dominant and recessive transmissions and the occurrence of less common X-linked mode (17). Additionally, increasing evidence for non-Mendelian inheritance associated with the co-occurrence of more than one genetic factor (i.e., digenic and oligogenic), seen in both KS and nIHH, has been reported (15, 18, 19). The cumulative effect of multiple deleterious variants in various genes impacts their interactions and eventually draws a phenotypical picture of IHH patients (20). Both classes of genes controlling GnRH neuron migration, development, and function and coding neuroendocrine pathways are strongly implicated in nIHH pathogenesis (15, 19, 21). Therefore, the broad clinical spectrum may result from oligogenicity, variable expressivity, inheritance pattern, or incomplete penetrance related to particular genes (15). Gene defects have been detected in approximately 2%–21% of subjects with nIHH and in approximately 50% of all IHH cases (15, 20, 22, 23). Pathogenic mutations in the gonadotropin-releasing hormone receptor gene (*GNRHR*) constitute the foremost cause of nIHH, particularly in familial cases (22). However, owing to the introduction of massive sequencing technologies into routine diagnostics, the efficacy of genetic cause identification rose significantly, and the importance of new IHH genes was revealed (19).

This study aimed to investigate the genetic background of nIHH in adult patients. In addition, the study examined the rates of pathogenic/likely pathogenic (P/LP) variants and variants of uncertain significance (VUS) detected using the next-generation sequencing (NGS) approach. The study also looked for the recurrence of those variants among patients with nIHH. The study aimed to further delineate a genotype–phenotype correlation, with special consideration of adult-onset nIHH (AO-nIHH).

## 2 Materials and methods

### 2.1 Patients and clinical evaluation

This study encompassed 67 unrelated Polish patients of Slavic origin (64 male and 3 female patients with an age range at genetic diagnosis of 16–46 years) diagnosed with nIHH. All patients underwent a complete physical and anthropometric examination, gynecological or andrological consultation with the sonographic assessment of the ovaries or testes, abdominal ultrasound examination, and pituitary magnetic resonance imaging (MRI). History of unilateral or bilateral cryptorchidism, micropenis, craniofacial anomalies (e.g., dental agenesis and cleft lip or palate), and hypogonadism in the family was obtained by a retrospective review of the patient’s medical records and from an interview with the patient. In the study group, there were 32 patients with AO-nIHH (48% of the tested subjects). Diagnosis of nIHH was based on the following criteria: (1) absent, delayed, or partial puberty or AO-nIHH; (2) reported and confirmed normal sense of smell; (3) clinical signs or symptoms of hypogonadism; (4) low or normal serum concentrations of luteinizing hormone (LH) and follicle-stimulating hormone (FSH) (typically less than 4 IU/L) and serum testosterone level <3.5 nmol/L in male patients or serum estradiol level <20 pg/mL in female patients; (5) otherwise normal anterior pituitary function; (6) no previous lesions of a sellar region in MRI or surgery of sellar region; and (7) normal serum ferritin concentration (24–26). The ability to maintain an average adult testosterone serum concentration after 6 months of withdrawal of hormone replacement therapy was used to determine the reversibility of IHH in male subjects (27). Patients who completed pubertal development with normal gonad size but demonstrated erectile dysfunction with loss of spontaneous nocturnal erections in male patients, loss of libido, and infertility occurring with decreased testosterone and normal/low gonadotropins, with an absence of known acquired IHH cause (weight loss, intensive exercise, chronic severe illness, etc.), were classified as AO-nIHH (28).

The concentrations of LH and FSH, growth hormone (GH), adrenocorticotropic hormone (ACTH), thyrotropin (TSH), prolactin (PRL), testosterone, estradiol, morning cortisol, dehydroepiandrosterone sulfate (DHEA-S), insulin-like growth 1 (IGF-1) factor, free triiodothyronine (fT3), and free thyroxin (fT4) were assessed using the Cobas 6000 system (Roche Diagnostics, Switzerland) and dedicated electrochemiluminescence sandwich immunoassay (ECLIA) kits provided by the manufacturer. The free testosterone was measured using the following formula: (FTI) = 100 × (total testosterone/SHBG). Hemochromatosis was excluded by testing serum ferritin levels (ECLIA, Cobas 6000 system, CV < 4.4%).

The clinical, biochemical, and smell testing methodology was described in the authors’ previous publication reporting patients with KS (29).

### 2.2 Genetic testing

NGS technology was used in the current study, and the gene panel was previously described and reported for KS patient cohorts (29). Briefly, the Ion Torrent Personal Genome Machine System (Ion PGM TM, Thermo Fisher Scientific, Inc. Waltham, MA, USA) was used, and a validated panel of 38 HH/CPHD genes was applied. Gene panel encompassed the following genes: *ADAM7, ANOS1, BMP2, BMP4, CHD7, FGF8, FGF17, FGFR1, GLI2, GNRH1, GNRHR, HESX1, HS6ST1, IGSF10, KISS1, KISS1R, LEP, LEPR, LHB, LHX3, LHX4, LRRIQ3, NSMF, NR0B1, OTX1, OTX2, PCSK1, PITX1, PITX2, PROK2, PROKR2, PROP1, POU1F1, SEMA3A, SOX3, TAC3, TACR3*, and *WDR11.* The sequences were primarily mapped to the human genome (GRCh37/hg19) using Torrent Suite™ software (version 4.0.2, Thermo Fisher Scientific, Waltham, MA, USA) and converted to GRCh38/hg38. Variants were assigned according to HGVS recommendations, and coding/genomic positions referred to the canonical transcripts. The pathogenicity estimation was made using Variant Effect Predictor (VEP) in Ensembl browser (19), MutationTaster2 (http://www.mutationtaster.org/), and PhenIX (http://compbio.charite.de/PhenIX/), and utilizing prediction from the following algorithms: FATHMM, CAD, PolyPhen2, SIFT, and MaxEntScan. Clinical databases HGMD (http://www.hgmd.cf.ac.uk) and ClinVar (https://www.ncbi.nlm.nih.gov/clinvar/) were also checked, and the frequency of variants was established using the GnomAD database (https://gnomad.broadinstitute.org/) with the minor allele frequency (MAF) of <1% as the filtering threshold. Alterations were classified according to updated ACMG guidelines [P/LP, VUS, and benign/likely benign (B/LB)] (30). Varsome (31) and InterVar (32) tools were used to determine the correctness of position estimation and HGVS nomenclature. Selected variants were subjected to *in silico* analysis to evaluate the effect of the change on protein architecture, considering its functionality and potential involvement in the manifestation of an abnormal phenotype.

Protein models were generated from FASTA files using homology modeling with Yasara 24.4.10 software (33), the PsiBLAST algorithm (34), and the Alphafold 3 server (35). The calculated structures, together with the model quality *Z*-scores, and PDB files were placed in the **Supplementary Materials**. Homology modeling in this version of the Yasara software uses, in addition to x-ray and NMR data (UniRef90H), the databases of three AI servers, i.e., Alphafold 2 (33), ESMFold (36), and OmegaFold. Models were aligned using the MUSTANG algorithm (37).

### 2.3 Institutional review board statement

The study was conducted according to the guidelines of the Declaration of Helsinki and approved by the Institutional Ethics Committee of Poznan University of Medical Science (1002/13, 5 December 2013; 990/15, 5 November 2015, 567/16, 5 May 2016).

### 2.4 Informed consent statement

Informed consent was obtained from all subjects involved in the study.

## 3 Results

In the study group, genetic defects were detected in 53 patients (79%). Four patients had cryptorchidism, three patients had micropenis, and one patient each had congenital adrenal hyperplasia (CAH), congenital adrenal hypoplasia, intellectual disability, arachnodactyly, facial bone dysplasia, and left renal agenesis. In the cohort of subjects presenting genetic abnormalities, 23 individuals were diagnosed with AO-nIHH, and the reversal form was found in only one patient. The majority of patients with AO-nIHH had no concomitant dysfunction or disorders. The detailed clinical data of the subjects were divided according to the onset and are presented in **Tables 1** and **2** (childhood vs. adult-onset, AO). No genetic defects were found in 14 patients (13 men and 1 woman). The AO-nIHH was confirmed in seven patients, whereas the reversal form was found in two patients with nIHH and no genetic defect. One case of horseshoe kidney, one case of strabismus, and one case of unilateral cryptorchidism were noted in patients with no genetic defect.

**Table 1:** Genetic test results and relevant clinical data in patients with childhood-onset nIHH with a genetic defect.

A. Patients with primary GnRH neuron migration/development defects											
Patient no./Gender	Stage	Gene	HGVS	Coding	ACMG	dbSNP	MAF	Accession	Variant type	Position	Clinical findings
1	GnRH neuron migration	CHD7	p.Lys850Gln	c.2548A>C	VUS	NR		NM_017780.4	Missense	Exon 8 of 38	Fibrolipomatosis of kidney
2	GnRH neuron migration	CHD7	p.Met2527Leu	c.7579A>C	Benign	rs192129249	0.00203	NM_017780.4	Missense	Exon 34 of 38	
3	GnRH neuron migration	CHD7	p.Arg2361Lys	c.7082G>A	VUS	rs777753993	0.0000371	NM_017780.4	Missense	Exon 33 of 38	Micropenis, oligodontia
4	GnRH neuron development	FGF8	–	c.445–62G>T	VUS	rs3218238	0.0000957	NM_033163.5	Intronic, splice site	Intron 5 of 5	
5	GnRH neuron development	FGF8	–	c.445–62G>T	VUS	rs3218238	0.0000957	NM_033163.5	Intronic, splice site, acc gained	Intron 5 of 5	Bilateral cryptorchidism
6	GnRH neuron development	FGFR1	p.Lys256Glu	c.766A>G	VUS	NR		NM_001174067.1	Missense	Exon 7 of 19	Reverse form of hypogonadism
**7**	**GnRH neuron development**	**FGFR1**	**p.Ile331Thr**	**c.992T>C**	**Likely pathogenic**	**rs121909633**	**0.000292**	**NM_001174067.1**	**Missense**	**Exon 8 of 19**	Arachnodactyly
	Pituitary dev. and signaling	NR0B1	p.Cys66Tyr	c.197G>A	VUS	NR		NM_000475.5	Missense	Exon 1 of 2	
	GnRH neuron development	HS6ST1	p.Ile89=	c.267C>T	Benign	rs1448339698	0.00000418	NM_004807.3	Synonymous	Exon 1 of 2	
**8**	**GnRH neuron development**	**FGFR1**	**p.Trp722CysfsTer35**	**c.2165_2166** **insCTGT**	**Pathogenic**	**NR**		**NM_001174067.1**	**Frameshift**	**Exon 17 of 19**	Preauricular skin tags
**9**	**GnRH neuron development**	**NSMF**	**p.Cys167Tyr**	**c.500G>A**	**Likely pathogenic**	**NR**		**NM_001130969.3**	**Missense**	**Exon 3 of 16**	
	Pituitary dev. and signaling	BMP2	p.Arg154Gln	c.461G>A	VUS	rs111675841	0.0000517	NM_001200.4	Missense	Exon 3 of 3	
10	GnRH neuron development	NSMF	p.His303=	c.909C>T	Benign	rs148475876	0.000201	NM_001130969.3	Synonymous	Exon 8 of 16	bilateral cryptorchidism
**11**	**GnRH neuron migration**	**PROKR2**	**p.Arg85Cys**	**c.253C>T**	**Pathogenic**	**rs141090506**	**0.000592**	**NM_144773.4**	**Missense**	**Exon 2 of 3**	Arnold Chari malformation
	**GnRH neuron development**	**FGF8**	**-**	**c.445–62G>T**	**Likely pathogenic**	**rs3218238**	**0.0000957**	**NM_033163.5**	**Intronic, splice site**	**Intron 5 of 5**	
12	GnRH neuron development	FGF17	–	c.-38G>A	VUS	rs147561706	0.00926	NM_003867.4	5’UTR	38 bp before transcription start site	Unilateral cryptorchidism
	GnRH neuron development	FGF8	–	c.445–62G>A	Benign	rs3218238	0.041	NM_033163.5	Intronic, splice site	Intron 5 of 5	
B. Patients with primary hypothalamic/pituitary development and signaling defects											
13	Pituitary dev. and signaling	GLI2	p.Asp1520Asn	c.4558G>A	VUS	NR		NM_005270.5	Missense	Exon 14 of 14	
	Pituitary dev. and signaling	GLI2	p.Met1352Val	c.4054A>G	VUS	NR		NM_005270.5	Missense	Exon 14 of 14	
**14**	**Pituitary dev. and signaling**	**GNRHR**	**p.Arg139His**	**c.416G>A**	**Pathogenic**	**rs104893842**	**0.000144**	**NM_000406.3**	**Missense**	**Exon 1 of 3**	
	Pituitary dev. and signaling	LHX4	p.Asp128=	c.384C>T	Benign	rs141139762	0.00864	NM_033343.4	Synonymous	Exon 3 of 6	
**15**	**Pituitary dev. and signaling**	**GNRHR**	p.Tyr283Thrfs*3	**c.846delC**	**Pathogenic**	**NR**		**NM_000406**	**Frameshift**	**Exon 3 of 3**	
	**Pituitary dev. and signaling**	**GNRHR**	**p.Leu141Pro**	**c.T422C**	**Pathogenic**	**NR**		**NM_000406**	**Missense**	**Exon 1 of 3**	
**16**	**Pituitary dev. and signaling**	**GNRHR**	**p.Arg139His**	**c.416G>A**	**Pathogenic**	**rs104893842**	**0.000144**	**NM_000406.3**	**Missense**	**Exon 1 of 3**	micropenis
	**Pituitary dev. and signaling**	**GNRHR**	**p.Arg262Gln**	**c.785G>A**	**Pathogenic**	**rs104893837**		**NM_000406.3**	**Missense**	**Exon 3 of 3**	
**17**	**Pituitary dev. and signaling**	**GNRHR**	**p.Arg139His**	**c.416G>A**	**Pathogenic**	**rs104893842**	**0.000144**	**NM_000406.3**	**Missense**	**Exon 1 of 3**	
	**Pituitary dev. and signaling**	**GNRHR**	**p.Trp206del**	**c.616_618del**	**Likely pathogenic**	**NR**		**NM_000406.3**	**In frame**	**Exon 2 of 3**	
**18 F**	**Hypothalamic signaling**	**KISS1**	**p.His90ThrfsTer57**	**c.268del**	**Likely pathogenic**	**NR**	**0.0000105**	**NM_002256.4**	**Frameshift original stop codon lost**	**Exon 3 of 3**	Myopia, intellectual disability
	Pituitary dev. and signaling	PROP1	p.Asn20Ile	c.59A>T	VUS	rs7445271	0.000064	NM_006261.5	Missense	Exon 1 of 3	
19 F	Pituitary dev. and signaling	LHX4	p.Arg208=	c.622A>C	VUS	rs1294068223	0.0000319	NM_033343.4	Synonymous	Exon 5 of 6	
**20**	**Pituitary dev. and signaling**	**NR0B1**	**p.Asn440Ile**	**c.1319A>T**	**Likely pathogenic**	**rs28935481**		**NM_000475.5**	**Missense**	**Exon 2 of 2**	Congenital adrenal hypoplasia
	GnRH neuron migration	CHD7	p.Lys850Gln	c.2548A>C	VUS	NR		NM_017780.4	Missense	Exon 8 of 38	
**21**	**Pituitary dev. and signaling**	**NR0B1**	**p.Ile373Ser**	**c.1118T>G**	**Likely pathogenic**	**NR**		**NM_000475.5**	**Missense**	**Exon 1 of 2**	Congenital adrenal hyperplasia, unilateral cryptorchidism
22	Pituitary dev. and signaling	OTX1	p.Gly19Val	c.56G>T	VUS	NR		NM_014562.4	Missense	Exon 3 of 5	
23	Pituitary dev. and signaling	OTX2	p.Arg127Pro	c.380G>C	VUS	rs199799627	0.00000795	NM_001270525.2	Missense	Exon 3 of 3	
24	Pituitary dev. and signaling	PCSK1	Intron 4 of 13	c.544–43T>G	VUS	NR		NM_000439.5	Intronic, splice site, acc gained	Intron 4 of 13	Left renal agenesis, right duplex kidney
25	Pituitary dev. and signaling	PROP1	p.Ser156Phe	c.467C>T	VUS	NR		NM_006261.5	Missense	Exon 3 of 3	
**26**	**Hypothalamic signaling**	**TACR3**	**p.Phe123LeufsTer2**	**c.369_372del**	**Pathogenic**	**0.00000398**		**NM_001059**	**Frameshift**	**Exon 1 of 5**	Bilateral cryptorchidism
	GnRH neuron migration	CHD7	–	c.1666–23C>T	VUS	NR		NM_017780.4	Intronic, splice site, acc gained	Intron 2 of 37	
	Pituitary dev. and signaling	HESX1	p.Met174Val	c.520A>G	VUS	NR		NM_003865.3	Missense	Exon 4 of 4	
**27**	**Hypothalamic signaling**	**TACR3**	**p.Trp275Ter**	**c.824G>A**	**Pathogenic**	**rs144292455**	**0.000307**	**NM_001059.3**	**Nonsense**	**Exon 3 of 5**	Micropenis
	**Hypothalamic signaling**	**TACR3**	**p.Phe123LeufsTer2**	**c.369_372del**	**Pathogenic**	**0.00000398**			**Frameshift**	**Exon 1 of 5**	
	Pituitary dev. and signaling	BMP4	p.Ser91Cys	c.272C>G	VUS	rs121912767	0.000182	NM_130851.3	Missense, splice site	Exon 3 of 4	
**28**	**Hypothalamic signaling**	**TACR3**	**p.Ala36GlnfsTer81**	**c.106delG**	**Likely** **Pathogenic**	**NR**		**NM_001059.3**	**Frameshift**	**Exon 1 of 5**	

F, female; ND, not detected. Pathogenic/Likely pathogenic variants were highlighted and written in bold.

**Table 2:** Genetic test results and relevant clinical data in patients with adult-onset nIHH with a genetic defect.

A. Patients with primary GnRH neuron migration/development defects											
Patient no./Gender	Stage	Gene	HGVS	Coding	ACMG	dbSNP	MAF	Accession	Variant type	Position	Clinical findings
**1**	**GnRH neuron migration**	**CHD7**	**p.Ser1146Gly**	**c.3436A>G**	**Likely pathogenic**	**NR**		**NM_017780.4**	**Missense**	**Exon 14 of 38**	
2	GnRH neuron migration	CHD7	p.Lys850Gln	c.2548A>C	VUS	NR		NM_017780.4	Missense	Exon 8 of 38	
3	GnRH neuron migration	CHD7	p.Ser103Thr	c.307T>A	Benign	rs41272435	0.0123	NM_017780.4	Missense	Exon 2 of 38	
4	GnRH neuron migration	CHD7	–	c.2377–4T>C	VUS	NR		NM_017780.4	Intronic, splice site	Intron 5 of 37	
	Pituitary dev. and signaling	NR0B1	p.Cys107=	c.321T>C	LB	NR		NM_000475.5	Synonymous, splice site	Exon 1 of 2	
5	GnRH neuron migration	CHD7	p.Glu1478=	c.4434A>G	VUS	NR		NM_017780.4	Synonymous, splice site, donor gained	Exon 19 of 38	scoliosis
**6**	**GnRH neuron migration**	**CHD7**	**p.Ser1146Gly**	**c.3436A>G**	**Likely pathogenic**	**NR**		**NM_017780.4**	**Missense**	**Exon 14 of 38**	
	GnRH neuron migration	ANOS1	–	c.542–6T>C	VUS	NR		NM_000216.4	Intronic, splice site	Intron 4 of 13	
	Hypothalamic signaling	TACR3	p.Phe78Leu	c.232T>C	VUS	rs767694452	0.0000159	NM_001059.3	Missense	Exon 1 of 5	
**7**	**GnRH neuron development**	**FGF8**	**p.Pro26Leu**	**c.77C>T**	**Likely pathogenic**	**rs137852660**	**0.00115**	**NM_033163.5**	**Missense**	**Exon 3 of 6**	
	GnRH neuron development	NSMF	p.Glu166Gly	c.497A>G	VUS	NR		NM_001130969.3	Missense	Exon 3 of 16	
8	GnRH neuron development	FGF8	–	c.445–62G>T	VUS	rs3218238	0.0000957	NM_033163.5	Intronic, splice site	Intron 5 of 5	
9	GnRH neuron development	HS6ST1	p.Arg367His	c.1100G>A	VUS	rs376288417	0.0000282	NM_004807.3	Missense	Exon 2 of 2	
	GnRH neuron development	FGF17	–	-	VUS	NR		NM_003867.4	5’UTR	61 bp before transcription start site	
10	GnRH neuron development	HS6ST1	p.Arg375Cys	c.1123C>T	VUS	rs559410305	0.0000687	NM_004807.3	Missense	Exon 2 of 2	
**11**	**GnRH neuron development**	**NSMF**	**p.Val123GlyfsTer69**	**c.365_366insA**	**Likely pathogenic**	**NR**		**NM_001130969.3**	**Frameshift, NMD**	**Exon 3 of 16**	
**12**	**GnRH neuron migration**	**SEMA3A**	**p.Asn271Asp**	**c.811A>G**	**Likely pathogenic**	**NR**		**NM_006080.3**	**Missense, splice site**	**Exon 8 of 17**	
	**GnRH neuron migration**	**SEMA3A**	**p.Ile110Val**	**c.328A>G**	**Likely pathogenic**	**NR**		**NM_006080.3**	**Missense, splice site**	**Exon 3 of 17**	
	**GnRH neuron development**	**FGF8**	**-**	**c.445–62G>T**	**Likely pathogenic**	**rs3218238**	**0.0000957**	**NM_033163.5**	**Intronic, splice site**	**Intron 5 of 5**	
	GnRH neuron migration	CHD7	p.His1891Tyr	c.5671C>T	VUS	NR		NM_017780.4	Missense, splice site	Exon 29 of 38	
13 F	GnRH neuron development	WDR11	p.Asn672Ser	c.2015A>G	VUS	NR		NM_018117.12	Missense	Exon 16 of 29	
	GnRH neuron development	WDR11	p.Ser621=	c.1863T>A	LB	NR		NM_018117.12	Synonymous	Exon 15 of 29	
14	GnRH neuron development	WDR11	p.Pro3Arg	c.8C>G	VUS	NR		NM_018117.12	Missense	Exon 1 of 29	
	Hypothalamic signaling	GNRH1	p.Phe61=	c.183C>T	Benign	rs6186	0.00524	NM_001083111.2	Synonymous	Exon 3 of 4	
B. Patients with primary hypothalamic/pituitary development and signaling defects											
**15**	**Pituitary dev. and signaling**	**BMP4**	**p.Gly249AlafsTer36**	**c.746delG**	**Pathogenic**	**NR**		**NM_130851.3**	**Frameshift**	**Exon 4 of 4**	
	GnRH neuron migration	SEMA3A	p.Asn153Ser	c.458A>G	VUS	rs139295139	0.00231	NM_006080.3	Missense	Exon 5 of 17	
**16**	**Pituitary dev. and signaling**	**BMP4**	**p.Gly249AlafsTer36**	**c.746delG**	**Pathogenic**	**NR**		**NM_130851.3**	**Frameshift**	**Exon 4 of 4**	
17	Pituitary dev. and signaling	GLI2	p.Leu1534Phe	c.4600C>T	VUS	NR		NM_005270.5	Missense	Exon 14 of 14	
18	Pituitary dev. and signaling	GLI2	p.Phe1581Ser	c.4742T>C	VUS	NR		NM_005270.5	Missense	Exon 14 of 14	right inguinal hernia
	GnRH neuron development	FGFR1	p.Asp166dup	c.495_497 dupTGA	LB	rs138489552	0.000272	NM_001174067.1	**In frame frameshift**	Exon 5 of 19	
19	Pituitary dev. and signaling	GLI2	–	c.1183–7T>C	VUS	NR		NM_005270.5	Intronic, splice site	Intron 8 of 13	
	Hypothalamic signaling		–	c.315A>G	LB	NR		NM_013251.4	Synonymous	Exon 6 of 7	
**20**	**Pituitary dev. and signaling**	**GNRHR**	**p.Arg139His**	**c.416G>A**	**Pathogenic**	**rs104893842**	**0.000144**	**NM_000406.3**	**Missense**	**Exon 1 of 3**	
21	Pituitary dev. and signaling	IGSF10	p.Val1983Asp	c.5948T>A	VUS	NR		NM_178822.5	Missense	Exon 7 of 8	
	GnRH neuron development	FGF8	p.Ile116=	c.348C>T	Benign	rs367853216		NM_033163.5	Synonymous	Exon 5 of 6	
	Pituitary dev. and signaling	OTX2	p.Ala253Thr	c.757G>A	VUS	rs139800030	0.0000239	NM_001270525.2	Missense	Exon 3 of 3	
22	Hypothalamic signaling	LEPR	p.Val754Met	c.2260G>A	VUS	rs150936702	0.000554	NM_002303.6	Missense	Exon 16 of 20	Facial bone dysplasia
23	Pituitary dev. and signaling	LHX4	p.Ser318Leu	c.953C>T	VUS	rs766990314	0.0000278	NM_033343.4	Missense	Exon 6 of 6	
	Pituitary dev. and signaling	OTX1	p.Ser183=	c.549G>T	LB	NR		NM_014562.4	Synonymous	Exon 5 of 5	
**24**	**Pituitary dev. and signaling**	**SOX3**	**p.Arg155AlafsTer26**	**c.462delG**	**Pathogenic**	**NR**		**NM_005634.3**	**Frameshift**	**Exon 1 of 1**	
	**Hypothalamic signaling**	**LEPR**	**p.Ile854AspfsTer35**	**c.2559_2560insG**	**Likely pathogenic**	**NR**		**NM_002303.6**	**Frameshift**	**Exon 18 of 20**	
	GnRH neuron migration	CHD7	p.Tyr2608=	c.7824T>C	VUS	NR		NM_017780.4	Synonymous	Exon 35 of 38	
25	Hypothalamic signaling	TACR3	p.Ile249Val	c.745A>G	LB	rs148732080	0.000148	NM_001059.3	Missense, splice site	Exon 3 of 5	
	GnRH neuron development	FGFR1	p.Asn847=	c.2541T>C	LB	rs1420466370	0.0000041	NM_001174067.1	Synonymous	Exon 19 of 19	

F, female; ND, not detected. Pathogenic/Likely pathogenic variants were highlighted and written in bold.

### 3.1 Genetic results

**Figure 1:**
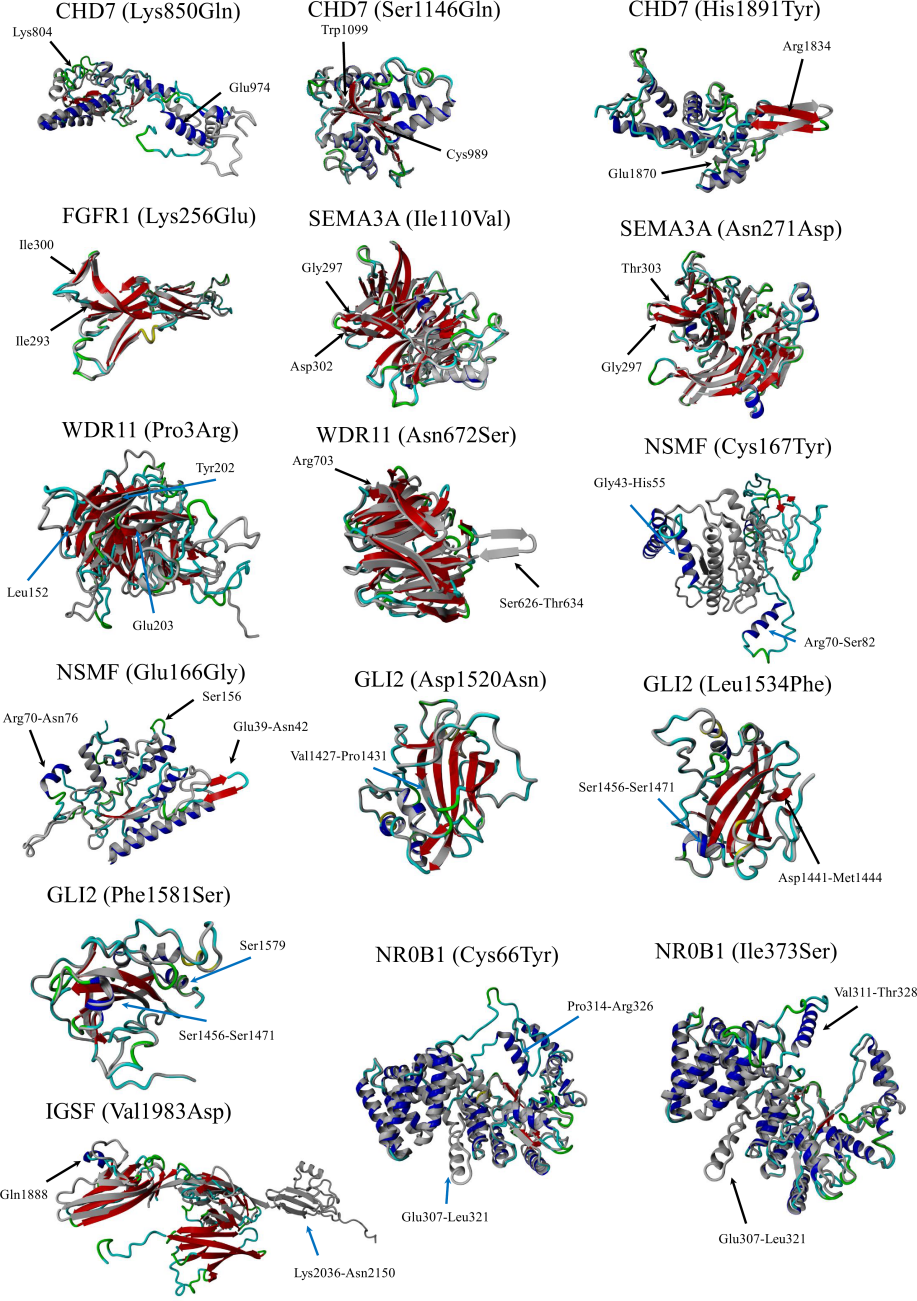
Simulated structures. The normal protein is colored gray, while the protein carrying a variant impacting structure is colored blue (coils) and red (β-pleated sheets). Protein models were generated using the Chimera v1.7 software.

**Figure 2:**
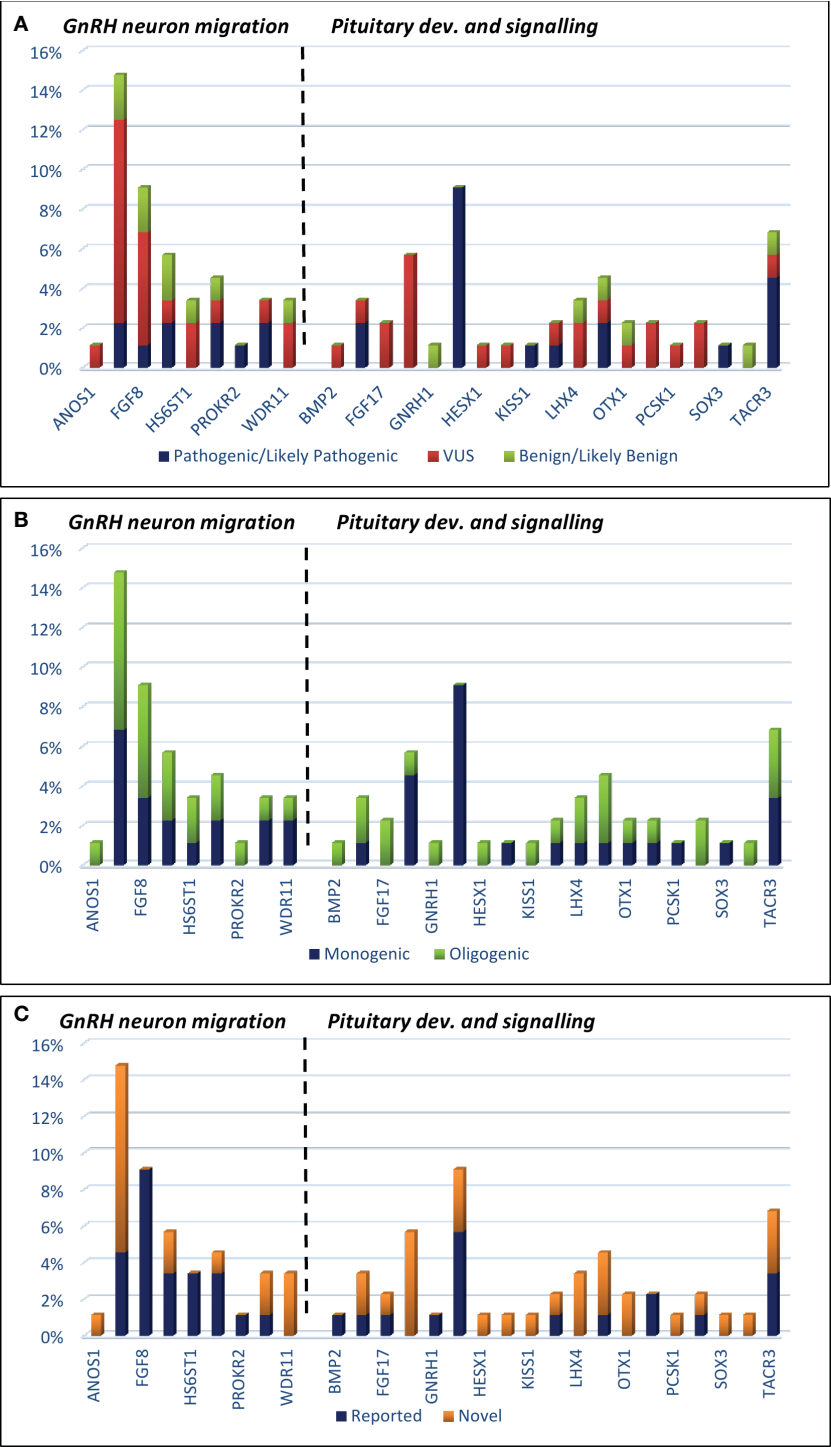
Histograms showing the prevalence of mutations among IHH patients, which are classified by three metrics: **(A)** mutation severity [ACMG criteria: pathogenic/likely pathogenic, VUS (variant of unknown significance), and benign/likely benign]; **(B)** oligogenicity; **(C)** novelty.

In the current study, the authors used the same strategy as in their work on patients with KS (29). Clinically relevant, identified variants were classified as those related to GnRH neuron development and migration (**Tables 1A**, **2A**) and those related to pituitary development and signaling (**Tables 1B**, **2B**). In total, defects in 27 genes were found in 53 patients with nIHH (79%), with at least one P/LP variant detected in 23 patients (34%) and VUS/B in 30 individuals (45%). Of the total 88 identified variants, 15 were classified as pathogenic (P), 16 as likely pathogenic (LP), 42 as VUS, and 15 as B/LB. The most frequently mutated genes presenting P/LP alterations were *GNRHR* (*n* = 5, 3 compound heterozygotes, 2 heterozygotes, and 3 novel and not reported); *TACR3* (*n* = 3; 1 compound heterozygote, 2 heterozygotes, and 3 novel), *CHD7* (*n* = 2, 2 novel), *FGFR1* (*n* = 2, 1 reported and 1 novel), *NSMF* (*n* = 2, 2 novel), *BMP4* (*n* = 2, 2 novel), and *NROB1* (*n* = 2, 1 reported and 1 novel). The hemizygous pathogenic variant was detected in the *SOX3* (*n* = 1; novel) and VUS variant in *ANOS1* gene (*n* = 1, novel). For 14 patients with nIHH, no causal or contributory variants were identified in the sequenced genes (21%). Finally, **Table 1** shows the genetic defects detected in 28 out of 35 patients with childhood-onset nIHH recruited in the study, and **Table 2** shows genetic variants in 25 out of 32 patients with AO-nIHH. In the childhood-onset nIHH group, significantly more P/LP variants were diagnosed (19) compared to the AO-nIHH group (11 defects). Monogenic P/LP variants were seen in 10 subjects (15%). Oligogenic defects with obligatory P/LP variant were detected in 13 patients (19%). In summary, 17 novel pathogenic variants, not previously reported for nIHH and affecting 10 genes (*CHD7, FGFR1, NSMF, SEMA3A, BMP4, GNRHR, NROB1, SOX3, LEPR*, and *TACR3* were detected in 17 patients). Concerning the recurrence of pathogenic variants, heterozygous changes *CHD7*:p.Ser1146Gly, *BMP4*:p.Gly249AlafsTer36, and *TACR3*:p.Phe123LeufsTer2 were noted in more than one patient. For reported pathogenic variants, recurrence was found for *GNRHR*:p.Arg139His (four patients). Surprising results were obtained regarding VUS variants. The total number of identified VUS changes represented half of all identified changes, emphasizing their possible importance for the evaluated phenotype. The recurrence was found for intronic change *FGF8*:c.445–62G>T (five patients), *CHD7*:p.Lys850Gln (three patients, VUS), and p.Ser1146Gly (two patients, LP). The high frequency of presented VUS variants among patients might be considered as likely pathogenic onwards. The overall most mutated gene-bearing VUS alterations were *CHD7* (nine patients), *FGF8* (five patients), and *GLI2* (five patients). Furthermore, those variants were intensively explored using the prediction of conformational changes, as presented in **Figure 1**. In summary, the applied approach allows identifying 40 variants in genes responsible for the development and migration of GnRH neurons and 48 alterations in genes contributing to the pituitary formation and pituitary/hypothalamic signaling. The histograms presenting the prevalence of mutations were classified by three metrics: severity of mutation (ACMG criteria: P/LP, VUS, and B/LB), oligogenicity, and novelty are shown in **Figure 2**.

### 3.2 Variant modeling

The simulated structures obtained from homology modeling are shown in **Figure 1**. The quality of models is represented by the *Z*-score parameter (**Table 3**). The accepted confidence scores were obtained for *CHD7* 750–1,000, 951–1,200, and 1,751–1,944 residues; *FGFR1* (133–350); *SEMA3A* (31–514); *NSMF* (2–220, p.Cys167Tyr); *IGSF10* (1,851–2,150); and *NR0B1*. The results were compared with the AlphaFold database and the confidence scores for the given residue range. While the *CHD7* protein was not present in the AlphaFold database, the rest of the protein models obtained similar confidence scores. For the *GLI2* domain 1,363–1,586, the *Z*-score was poor, although better than the AlphaFold structure for which only loops were present, and no structural information was achievable. The abbreviated structures were checked and presented using Chimera software. All calculated structures, including PDB structures, were placed in the **Supplementary Materials**.

**Table 3:** Protein models’ overall *Z*-score parameters and comparison with AlphaFold database.

Protein	Range	Variant	*Z*-score native	*Z*-score variant	Overall Yasara model quality	AlphaFold confidence score for native protein	Comment
CHD7	750–1000	p.Lys850Gln	-1.938	−2.032	Satisfactory	Not present	
CHD7	951–1200	p.Ser1146Gly	−1.216	−1.222	Satisfactory	Not present	
CHD7	1751–1944	p.His1891Tyr	−3.104	−1.802	Poor/Satisfactory	Not present	
FGFR1	133–350	p.Lys256Glu	−0.865	−1.062	Good	Low–very high	
SEMA3A	31–514	p.Ile110Val	−1.327	−1.305	Satisfactory	Very high	
SEMA3A	31–514	p.Asn271Asp	−1.327	−1.491	Satisfactory	Very high	
WDR11	1–394	p.Pro3Arg	−2.493	−2.692	Poor	Low–very high	
WDR11	551–800	p.Asn672Ser	−2.393	−2.380	Poor	Low–very high	
NSMF	2–229	p.Glu166Gly	−2.736	−2.756	Poor	Very low–low	
NSMF	2–220	p.Cys167Tyr	−2.736	−1.590	Poor/Satisfactory	Very low–low	
GLI2	1363–1586	p.Asp1520Asn	−3.46	−3.718	Poor	Very low–low (loops only)	FoldX from p.Asp1520Asn to native
GLI2	1363–1586	p.Leu1534Phe	−3.46	−3.747	Poor	Very low–low (loops only)	FoldX from native to p.Leu1534Phe
GLI2	1363–1586	p.Phe1581Ser	−3.46	−3.387	Poor	Very low–low (loops only)	FoldX from native to p.Leu1581Phe
IGSF10	1851–2150	p.Val1983Asp	−1.898	−1.461	Satisfactory	Confident	
NR0B1	1–470	p.Cys66Tyr	−1.771	−1.659	Satisfactory	Very low–confident	
NR0B1	1–470	p.Ile373Ser	−1.891	−1.529	Satisfactory	Very low–confident	

### 3.3 Genotype–phenotype correlation

In patients with nIHH, P/LP defects were found more often in genes related to hypothalamic–pituitary signaling pathways than in GnRH neuron migration/development genes. Two patients with concomitant adrenal diseases (CAH and congenital adrenal hypoplasia) were found to have LP-type mutations in the *NR0B1* gene. In the group of women studied (five patients), the LP variant was detected in one of them within the *KISS1* gene. In addition to HH, she was also diagnosed with intellectual disability.

There was a greater diversity of genetic variants in patients with AO-nIHH than in the group with prepubertal or peri-pubertal timing of diagnosis. Only in the latter group were defects in the FGFR1, NR0B1, and TACR3 genes present, and the *GNRHR* defect was prevalent (**Figure 2**).

## 4 Discussion

Unlike the KS form, the normosmic form of IHH is sporadically manifested with congenital nonreproductive abnormalities. Regarding the fact that multiple genes contribute to both forms, the reasonable explanation for phenotypical variability would be the nature and deleteriousness of variants and their impact on protein functionality. This study included extensive genetic diagnostics performed on the largest Polish nIHH patient group to date. Assuming a more complex and severe KS phenotype compared to nIHH, one could expect that stronger pathogenic alterations will play a key role in KS, and nIHH forms will result from milder, less harmful changes. This situation was well documented for *CHD7* gene in patients with IHH and CHARGE syndrome (38). Unfortunately, strong pathogenic variants are seen for both KS and IHH. Therefore, their functional impact is difficult to estimate, while the action of extra-genetic factors cannot be excluded. The phenotypic complexity is further evidenced by new reports of oligogenicity and a growing role of causatives of accumulated milder variants in a single patient (19, 28, 39). A thorough bioinformatic analysis of data obtained in NGS and dedicated genetic panels (considering the clinical picture) is necessary. In patients with childhood-onset IHH, indicative of a more severe form, a greater number of LP/P defects were observed compared to AO-IHH. Patients with a more severe form of the disease, which manifests earlier, tend to exhibit more significant genetic defects (19, 28, 39).

The efficacy of setting up molecular diagnosis in patients with nIHH was higher than expected, approximately 20% according to literature and involved 34% of the examined cohort (P/LP variant identified in patients) (23). However, this number is promising since it is close to the rate of 40% for successful diagnosis for patients with KS (6, 7). The data confirm that known HH genes have an undisputable role in representing a solid foundation for comprehensive diagnostics. Focusing in detail on those genes and expanding analyses for their non-coding or regulatory sequences (i.e., splicing regulation) may benefit in leveraging mutation detection rate as evidenced by this study. The VUS call is made for the majority of clinically relevant variants since their direct deleterious effect on protein is predictive but difficult to prove (functional experiments required). Apart from that, VUS still meets important ACMG criteria (PM2 and BS2) of much higher frequency of such alterations in the patient group compared to the control or the combined general population. The contribution of VUS changes to the phenotype is further discussed in this manuscript.

Oligogenicity occurred in 19% of the patients in the current study, which is close to some data published so far (40). According to recent data, the molecular diagnosis was set for 34% of patients, which is more than expected for this cohort (23). However, it should be considered that publications differ in the number of genes studied. The most frequently mutated genes for P/LP variants were *GNRHR* (eight changes) and *TACR3* (four changes), regarding genes contributing to pituitary development and signaling (a total of 19 alterations). In the case of genes playing a role in GnRH neuron migration, the frequencies were shared between multiple genes: *CHD7, FGFR1, NSMF*, and *SEMA3A* (two P/LP variants per gene, a total of 10 changes in this group). Therefore, the first striking conclusion that can be drawn is a higher contribution of genes related to the pituitary with a ratio of 1.5/1. Another notable finding of this research is the contribution of VUS variants. Compared to P/LP variants (29 identified), the overall frequency was higher (44 changes identified), and comparing both pathways (GnRH neuron migration and pituitary signaling), it was equally distributed (22/22). This fact raises the question of the clinical relevance of such variants, particularly regarding missing heritability in molecular diagnostics of patients with nIHH and obvious recurrence in the studied cohort. Recent data have confirmed the contribution of copy number variants (CNVs) in the etiopathogenesis of IHH (41). In the authors’ previous work on CPHD, they also confirmed the presence of such alterations in the genetic landscape of the disease (42). Therefore, the current study examined coding sequences of genes and sought long homozygous regions that would suggest potential allele deletions and prompt microarray scanning to be performed. Unfortunately, no such regions were found. It is agreed with Stamou et al. that these changes are possible but represent minor causes among patients with nIHH and present low prevalence (20). Taking into account all the issues mentioned, the authors took a closer look at selected genes where rare nucleotide variants were identified.

### 4.1 Hypothalamic/pituitary signaling and development

As expected, the authors noted the significant contribution of *GNRHR* variants, as this gene plays an essential role in IHH. Strikingly, all the identified changes were pathogenic and followed typical recessive transmission. The most familiar change was *GNRHR*: p.Arg139His (four patients) that appeared in four out of five patients presenting the nIHH phenotype. This variant is rare (MAF = 0.000114) and showed up in compound heterozygosity with other pathogenic changes: p.Trp206del and reported p.Arg262Gln (seven studies) (43). The mutation has so far been identified in 11 individuals with nIHH and may therefore represent a founder mutation in the Polish cohort. The three novel alterations among patients were truncating (p.Tyr283Thrfs*3; p.Trp206del) and missense (p.Leu141Pro). Surprisingly, no VUS variants were reported in this gene. Another critical gene in this cohort was *TACR3*. Similarly, as in GnRHR, biallelic loss-of-function mutations result in clinical consequences. In the study, three P/LP alterations (all truncating, two novel), one VUS and benign change (both missense and rare MAF = 0.0000159 and MAF = 0.000148, so far not reported for nIHH) were identified. The frameshift change p.Phe123LeufsTer2 was recurrent, and only one individual in the population database was found (MAF 0.00000398). Although the pathogenicity of detected variants seems convincing, biallelic deleterious changes were found not for all patients. Homozygosity or compound heterozygosity was tracked for three out of five patients regarding *GnRHR* and only one out of five for *TACR3*. Assuming AR inheritance, conclusive diagnostics for such patients cannot be made. These data suggest the need to expand sequencing for intronic alterations or focus more on existing rare VUS changes, which might have a decisive role in expressing phenotype.

Another justification for this strategy is the low oligogenicity rate and strong expectation for a typical monogenic background. One more mutated gene that did not escape the authors’ attention was transcription factor *GLI2*. Five novel VUS alterations were identified (four missense and one intronic) that were predicted to be deleterious according to applied algorithms, affecting the distal part of a gene, particularly in an evolutionarily conservative region in the vicinity of a carboxy-terminal domain responsible for transcriptional activation. The more detailed structure analysis the authors applied confirmed earlier predictions (**Figure 1**). Hitherto, the *GLI2* gene was not linked to nIHH but was evidenced to impact pituitary function in hypopituitarism. The Gli2 protein contributes to Shh signaling in pituitary embryogenesis, expressed in the ventral diencephalon, where it induces *Bmp4* and *Fgf8* expression, and in the oral ectoderm, it induces pituitary progenitors (44). This finding encouraged the authors to look closely at coding sequences in the mentioned interacting genes. Pathogenic variants were also found for both, emphasizing the importance of pituitary development processes and their proper functioning. For *BMP4*, recurrent truncation p.Gly249AlafsTer36 (so far not reported) was identified. The second reported change was p.Ser91Cys (rs121912767, MAF = 0.000182). This change was evidenced as causative for kidney anomalies and supported by functional studies (45, 46), therefore meeting the criteria for a pathogenic call (PM2, PS3, PP3, and PP5), although the ClinVar database interpreted this variant as VUS due to conflicting reports. Since variants in the *TACR3* gene were also shown in a patient with nIHH who had this defect, it is speculated that it had not had a direct impact on the disease, maintaining its uncertain significance.

Two different variants of the *NR0B1* gene have been detected in patients with nIHH and concomitant adrenal dysfunction (congenital adrenal hypoplasia and CAH). The first one, missense p.Asn44Ile, had already been reported in X-linked primary adrenal insufficiency with associated IHH (47). Another novel missense variant, p.Ile373Ser, was present in a patient with nIHH and CAH. Single cases in the literature describe patients with *NR0B1* and CAH (48).

### 4.2 GnRH neuron migration/development

A further gene whose expression is induced by *GLI2* and turned out to be mutated in the nIHH cohort under study was *FGF8*. The protein belongs to a broad family of fibroblast growth factors and, as the ligand for the *FGFR1* receptor, is directly involved in GnRH neuron migration (49). Data from the authors’ patients delivered pathogenic missense variant p.Pro26Leu (reported in patients with KS) and rare intronic variant c.445–62G>T (MAF = 0.0000957). This nucleotide position is conservative evolutionarily and was registered as a candidate for hypospadias (50) (HGMD CS072207) in its more common form c.445–62G>A (MAF = 0.04; in the cohort of the current study, only one patient). Surprisingly, five other patients with nIHH presented the G>T variant, which is predicted to affect splicing according to the NNSplice algorithm (SS acceptor gained). The alteration was estimated as VUS since interpretation relies on splice site prediction, and change might be population-specific. It is worth noting that all five patients with nIHH are sporadic, not related, and non-consanguineous. The authors also checked genomic data from testing other individuals (i.e., KS cohort, controls, and 300 chromosomes), and a variant was not found (29). The gene coding receptor for fibroblast growth factor (*FGFR1*) revealed abnormalities for five patients (two P/LP, one VUS, and two B/LB rare variants). The pathogenic variants p.Trp722CysfsTer35 and VUS p.Lys256Glu have not yet been reported. The missense change p.Ile331Thr was reported in patients with nIHH and is rare (MAF = 0.000292); however, in ClinVar, conflicting data on pathogenicity is assigned. This gene, in multiple studies, is regarded as the most frequently mutated and major IHH player affecting both KS and nIHH, sometimes even linked to the pleiotropic presentation (51). An evidenced contribution of other genes involved directly in the neurogenesis of the olfactory bulbs or neural crest cell guidance like *PROKR2* and *SEMA3A* in nIHH phenotype was noted for one (*PROKR2*:p.Arg85Cys) and two patients, respectively (*SEMA3A*: p.[Asn271Asp]/[Ile110Val]; *SEMA3A*:p.Asn153Ser). These findings strengthen the importance of testing such genes routinely, even though significant variants showed higher frequency in the Asian population (52).

The unclear role of the *NSMF* gene (formerly *NELF*) and its co-occurrence with oligogenic cases is still disputable (53). The current study identified four *NSMF* variants (two P/LP, one VUS, and one B/LB), and three of them were novel. The pathogenic change p.Val123GlyfsTer69 was a single monogenic defect, whereas two others, p.Cys167Tyr (LP) and p.Glu166Gly (VUS), appeared in combination with variants in *FGF8* and *BMP2*. Further studies are required to determine the importance of the *NSMF* gene in the pathogenesis of nIHH.

Finally, the gene that deserves detailed analysis is *CHD7*. Multiple families carrying *CHD7* mutations in the AD form demonstrated a broad phenotypic variability, including CHARGE syndrome (OMIM 214800) and IHH (54). However, loss-of-function or truncating mutations in this gene are expected to be linked more frequently to the syndromic phenotype (CHARGE and KS) and lower frequency in nIHH (38, 55). Some authors even suggest that patients with milder phenotypes underwent insufficient exhaustive clinical examination (olfactometry, olfactory bulb MRI, deafness and outer-ear abnormalities, and cardiac problems), and milder syndromic symptoms might be simply overlooked (56, 57). Striking pleiotropy, like those observed in *FGFR1*, was noted for selected pedigrees as patients harboring identical mutation presented various phenotypical spectrums of KS, nIHH, or isolated anosmia (57). The assessment of pathogenicity for such genes as *FGFR1* and *CHD7* is therefore challenging, regarding their structural conformational complexity and the resulting abundance of involvement in multiple developmental processes. Its principal role includes neural crest cell migration impacting craniofacial bone formation or the development of a peripheral nervous system and cardiac structures (58).

Additionally, *CHD7* is impacting phenotype indirectly via regulating multiple neurodevelopmental genes, i.e., *SEMA3A*, an important contributor to olfactory neuronal guidance. In the current study, pathogenic variants were identified in two patients (recurrent missense p.Ser1146Gly) and VUS variants were identified in another nine patients (three individuals with p.Lys850Gln recurrent missense, and missense/splice site intronic for others). Therefore, the observation of a higher frequency of non-truncating changes presenting partial loss of function or diminished residual activity of the protein, resulting in nIHH, seems to be confirmed in the cohort of the current study. However, the number of identified VUS alterations addresses an essential question of their clinical utility or relevance and further directives for molecular diagnostics in patients presenting with nIHH.

### 4.3 Unresolved role of VUS variants and oligogenicity in nIHH

Multiple recent reports have presented robust, comprehensive, reasonably designed methodological approaches and testing strategies utilizing genomic tools like whole exome sequencing and whole genome copy number analysis (41). Although the applied methods have been proven to be efficient, they have not resulted in the identification of new genes that would explain missing heritability. The number of genes so far reported (~60) constitutes a core target for the nIHH phenotype, and it appears that it will not be raised dramatically. Despite some difficulties related to various gene disruption mechanisms (RSVs and CNVs), most researchers have applied a similar and efficient methodology. The definitive molecular diagnosis is still unsuccessful for over half of all patients with nIHH. In the current study, 44 VUS alterations were identified, and this number is higher than the total number of P/LP changes (28), which is rather expected and in agreement with other reports. Considering VUS for molecular diagnosis, the diagnostic yield would increase dramatically, but according to ACMG criteria and recommendations, that may also result in incorrect diagnosis and further improper genetic consultation. Therefore, all those criteria for pathogenicity estimation should comply rigorously. However, there are also several important reasons why it should be included in scientific reports. First, the striking observation is that most of the VUS found in the current study (55%) have not been reported. They usually met moderate ACMG principles: PM2 (absent from controls and population datasets), PM6 (assumed *de novo*, but without confirmation), sometimes for familial cases PP1 (cosegregation with disease among family members), PP2 (missense variants in genes as the common cause of disease), and finally PP3 (computing deleterious effects, conformation, conservation, splice, etc.). If the variant is identified and confirmed by others in a similar cohort of patients, it falls under the PS4 rule (strong evidence, prevalence in affected, and significantly increased compared to controls), which automatically shifts the estimation from VUS to likely pathogenic. Secondly, reporting VUS changes can prompt researchers to perform a functional evaluation in order to recapitulate the disease phenotype and encounter another strong ACMG rule, PS3 (evidence of damaging effect in functional studies). Missense variants often require individual evaluation because of unpredictable effects on protein function or a selected pathway (not necessarily a primary pathway) in case of multi-functional genes (i.e., *CHD7*). Recently, a comprehensive functional study on KS-linked genes *FGFR1*, *HS6ST1*, *SOX9/10*, and *CHD7* was conducted. Using machine learning-based predictions and functional knock-outs, the authors evidenced convergence in signaling pathway and contribution to the same processes via various activities (59). Moreover, a significant burden is related to intronic changes since such variability is usually ignored due to a lack of convincing evidence (difficulties in estimation pathogenicity, limited access to algorithms predicting disruption of splice/regulatory sites, and their reliability). A similar problem is addressed in the estimation of oligogenicity consequences, where a synergistic effect is postulated. Here, it is even challenging to design proper functional studies, and the only possible line of evidence is the systematic reporting of genetic background in conclusive cohorts of patients.

### 4.4 Limitations

Potential limitations of the study include its limited sample size and the use of NGS and a 38-gene targeted panel. Ideally, a whole genome sequencing approach and large, international, and interethnic groups should be used. The groups of patients with nIHH in other studies were of comparable size. Most studies have focused either holistically on patients with IHH or selectively on KS form. There are few studies on the genetic basis of nIHH that have such extensive NGS diagnostics and a dedicated gene panel. There are studies in which the groups of patients with hypogonadotropic hypogonadism without olfactory disorders are larger, but they probably included patients with hypogonadism secondary to, for example, obesity, among others. The selection of patients for the current study was very thorough and aimed at qualifying patients whose genetic background of hypogonadism was most likely, after excluding secondary causes.

## 5 Conclusions

Genetic screening should be offered in patients with nIHH, with special consideration for adult-onset forms. Careful qualification of the IHH patient for genetic testing seems to be crucial. In summary, the molecular diagnostics was successful for 34% of individuals with nIHH, and the applied strategy relying on clinically targeted sequencing is robust. The principal role of the neuroendocrine pathway and related genes like *GNRHR1*, *TACR3*, or *NR0B1* is undisputed. The neurodevelopmental pathway, mainly associated with the migration of GnRH neurons, is becoming increasingly important. New insights into multifunction genes like *CHD7, FGFR1, NSMF*, *SEMA3A*, and *FGF8* were made. A total of 29 pathogenic variants were added to the etiopathogenesis of IHH. The study also focused on the underestimated role of VUS variants, particularly those presenting recurrence, and genes that were found to be commonly affected by such variants as *CHD7*, *FGF8*, or *GLI2*.

Further analyses are needed to delineate the causes and mystery of the genetic landscape in patients with nIHH with unresolved disease backgrounds. It is important to have a proper interpretation of Mendelian and non-Mendelian inheritance patterns, as well as oligogenic interactions between genes, to gain an advanced understanding of the nature of nIHH and provide effective genetic counseling. Further development of the availability of targeted molecular diagnostics of nIHH is required.

## Data availability statement

The raw data supporting the conclusions of this article will be made available by the authors, without undue reservation.

## Ethics statement

The studies involving humans were approved by Institutional Ethics Committee of Poznan University of Medical Science (1002/13, 5th December 2013; 990/15, 5th November 2015, 567/16, 5th May 2016). The studies were conducted in accordance with the local legislation and institutional requirements. The participants provided their written informed consent to participate in this study.

## Author contributions

MK: Conceptualization, Funding acquisition, Investigation, Writing – original draft, Writing – review & editing. BB: Conceptualization, Data curation, Formal Analysis, Writing – original draft, Writing – review & editing, Investigation, Methodology, Software. MRa: Investigation, Writing – review & editing. AD: Investigation, Writing – review & editing. MT-M: Writing – review & editing, Investigation. KS: Writing – review & editing, Investigation, Software, Visualization. AP: Writing – review & editing, Investigation, Software, Visualization. EW: Writing – review & editing, Investigation. AH-D: Writing – review & editing, Investigation. MRu: Writing – review & editing, Investigation, Supervision. KZ: Writing – original draft, Writing – review & editing, Investigation.
